# Effective Interventions to Prevent Breastfeeding-Related Nipple-Areolar Lesions: A Systematic Review

**DOI:** 10.3390/ijerph23020189

**Published:** 2026-01-31

**Authors:** Ana Chagas, Fernanda Moura, Monise Bispo, Lays Medeiros, Isabelle Costa, Rhayssa Araújo

**Affiliations:** Department of Nursing, Federal University of Rio Grande do Norte, Natal 59078-900, RN, Brazil; larysagaldino@gmail.com (A.C.); fernandaevellyn22@gmail.com (F.M.); monise.bispo.078@ufrn.edu.br (M.B.); laysp_medeiros@hotmail.com (L.M.); isabelle.fernandes@ufrn.br (I.C.)

**Keywords:** nipples, wounds and injuries, breastfeeding, primary prevention, nursing

## Abstract

**Highlights:**

**Public health relevance—How does this work relate to a public health issue?**

- Nipple-areolar lesions are a public health problem because they compromise breastfeeding, leading to maternal suffering, early weaning, and negative impacts on infant health and key breastfeeding indicators.
- They also increase avoidable demands on health services and strain on health systems, especially in vulnerable contexts, making their prevention essential for efficient, equitable, and sustainable care.

**Public health significance—Why is this work of significance to public health?**

- This study synthesizes evidence on effective interventions to guide breastfeeding promotion, prevention, and care, supporting safer, more effective, and evidence-based clinical practice.
- By preventing injuries, reducing pain, and improving the breastfeeding experience, it strengthens maternal autonomy, supports sustained breastfeeding, reduces childhood illness and healthcare costs, and contributes to global maternal-child health goals such as the SDGs.

**Public health implications—What are the key implications or messages for practitioners, policymakers, and/or researchers in public health?**

- For practitioners, the evidence shows that preventing nipple injuries must be a core element of breastfeeding care, with early risk identification, active guidance on positioning and latching, and continuous follow-up to reduce pain and improve maternal and child outcomes.
- For policymakers and researchers, the findings support strengthening education strategies, care protocols, and preventive technologies, guiding future research and policies that improve care quality and promote sustainable breastfeeding support.

**Abstract:**

This study synthesizes the evidence on effective interventions for the prevention of breastfeeding-related nipple-areolar injuries. A systematic review was performed and guided by the evidence synthesis manual of the Joan Briggs Institute, carried out in six databases, with only intervention studies. Interventions with a positive outcome for the prevention of nipple-areolar lesions were considered effective. Methodological quality was assessed by the Grading of Recommendations Assessment, Development and Evaluation. The final sample of 14 articles found the following to be effective strategies: educational practices (simulations and demonstrations of the breastfeeding technique, with a qualified professional or by video) and the use of peppermint (aqueous solution or gel), extra virgin olive oil, honey, guaiazulene ointment, and venix caseosa. Each intervention was used in specific situations and ways, which should be considered for use in clinical practice. The interventions discussed can help prevent nipple-areolar lesions and breastfeeding difficulties, encouraging breastfeeding.

## 1. Introduction

The World Health Organization (WHO) recommends exclusive breastfeeding for the first six months of life, continuing until at least two years of age, alongside the introduction of complementary foods [1]. This practice is the best choice for the growth and development of infants due to the nutritional and immunological properties of breast milk, as well as the opportunity to establish and strengthen the emotional bond between mother and baby [2].

Additionally, breastfeeding is essential for the maturation of the infant’s physiological systems, as it provides immunological, nutritional, and biological compounds that support early-life development [3].

Breastfeeding plays a critical role in the maturation of the infant’s physiological systems, contributing to immune protection, gastrointestinal development, and neurocognitive outcomes, as well as reducing the risk of infectious diseases and long-term chronic conditions. For mothers, it offers protection against breast and ovarian cancer, helps prevent postpartum hemorrhage, and reduces the risk of chronic diseases such as type 2 diabetes and cardiovascular conditions [4].

Despite its importance, breastfeeding rates in the Americas, for example, only show about 38% of babies under six months of age who are exclusively breastfed, which is below the target of at least 70% by 2030 [1,5]. Various factors contribute to this, particularly in the first weeks of the postpartum period when the most common breast complications occur, such as the development of nipple-areolar lesions. Although highly prevalent, these lesions should not be overlooked [6,7].

Nipple-areolar lesions are characterized by alterations in the nipple-areolar skin, identified by changes in color, thickness, fluid content, or tissue loss. In most cases, these lesions are associated with early weaning due to pain and difficulties in achieving proper latch-on by the infant [8,9,10]. These lesions typically persist for an average of seven days postpartum, with healing time depending on their severity and extent, potentially lasting one to two weeks. They affect approximately 58% of postpartum women, highlighting their high prevalence in this population [11].

The etiology of nipple-areolar lesions is multifactorial and involves maternal, infant-related, and breastfeeding management factors. Commonly reported causes include improper positioning and latch, ineffective sucking patterns, prolonged or frequent feeding without adequate milk transfer, nipple engorgement, and excessive friction or moisture on the nipple-areolar complex. Infant anatomical conditions that compromise oral function also play a significant role. In particular, a short sublingual frenulum (ankyloglossia) may restrict tongue mobility, impair effective latch and milk extraction, and increase mechanical stress on the nipple, thereby contributing to nipple pain and tissue damage in breastfeeding women [9,10,11].

Given the high prevalence of nipple-areolar lesions and their negative impact on breastfeeding continuation, identifying effective preventive interventions is essential to support sustained breastfeeding and maximize its health benefits for both mothers and infants. In this process, it is important to highlight that in the context of areola-nipple lesions related to breastfeeding, preventive strategies can be conceptually divided into primary prevention, which aims to avoid the onset of tissue damage, and secondary prevention, focused on the management of already established lesions.

However, the existing literature has predominantly emphasized treatment approaches, while evidence specifically addressing primary prevention is still limited. Therefore, this study focuses exclusively on primary prevention interventions, defined as strategies implemented before the development of areola– nipple lesions to reduce their occurrence.

Thus, the objective of this study is to synthesize the evidence on effective interventions for preventing nipple-areolar lesions caused by breastfeeding.

## 2. Methods

### 2.1. Study Design, Period, and Location

This is a systematic review of efficacy, conducted using a self-developed protocol and guided by the Preferred Reporting Items for Systematic Reviews and Meta-Analyses (Supplementary Material PRISMA checklist) [12] and the Joanna Briggs Institute Manual for Evidence Synthesis (Aromataris) [13].

The systematic review was carried out in Natal, Rio Grande do Norte, Brazil, from June 2021 to July 2025. Database searches were conducted between September 2024 and July 2025.

### 2.2. Population or Sample and Inclusion and Exclusion Criteria

The guiding research question followed the PICO strategy (Population, Intervention, Comparison, and Outcome) (PRISMA) [12]. The defined population included lactating women at any stage of breastfeeding. Interventions encompassed any actions taken to prevent nipple-areolar lesions caused by breastfeeding, regardless of type, frequency, or intensity. Regarding comparison, since this review aims to address a broader question, all existing alternative interventions were considered (placebos, standard care, other preventive strategies, or even no intervention). The outcome was the prevention of nipple-areolar lesions caused by breastfeeding, regardless of how it was measured. Thus, the research question was formulated as follows: Which interventions are effective in preventing nipple-areolar lesions in lactating women?

Regarding eligibility criteria, this review included interventional studies, original articles available in full text, and studies addressing preventive care for nipple-areolar lesions related to breastfeeding in lactating women. Studies whose primary objective was the treatment or management of already established nipple-areolar lesions were excluded.

### 2.3. Study Protocol

This study was registered in the International Prospective Register of Systematic Reviews (PROSPERO), an international database of systematic reviews (identification: CRD42021278258).

The searches were carried out using the CAPES (Coordination for the Improvement of Higher Education Personnel) platform through CAFe (Federated Academic Community) access, in the Medical Literature Analysis and Retrieval System On-line/National Library of Medicine (Medline/Pubmed), Scopus, Cumulative Index to Nursing and Allied Health Literature (Cinahl), Web of Science, Cochrane and Latin American, and Caribbean Health Sciences Literature (Lilacs) databases.

The descriptors were selected from DECs/Mesh Terms in English and Portuguese. Different search strategies were tested in order to find as many potential studies as possible.

Thus, the databases Web of Science, Cochrane, and Lilacs were used: Nipple* (OR Nipple trauma OR nipple soreness OR sore nipples OR nipple fissure OR nipple cracked OR nipple cracks) AND Wound and injuries AND Breastfeeding AND Primary prevention (OR prevention) AND Clinical trial NOT Treatment. In the grassroots Medline/Pubmed, Cinahl e Scopus utilizou-se: Nipple* (OR Nipple trauma OR nipple soreness OR sore nipples OR nipple fissure OR nipple cracked OR nipple cracks) AND Wound and injuries AND Breastfeeding AND Primary prevention (OR prevention).

Each database obtained a different search strategy due to the application of filters. In the Medline/Pubmed database, the following filters were used: free full text, comparative study, clinical trial, controlled clinical trial, randomized controlled trial, teen (13–18 years), adult (19+ years) e female. In Cochrane, the following filters were used: Trials; in Scopus, open access; Web of Science, open access and article. In Cinahl, the following filters were used: full text filter; Lilacs, not filter.

### 2.4. Analysis of Results and Statistics

The data from the selected articles was extracted, entered, and organized in a self-made spreadsheet on the Excel 2016 platform with a description of identification (base, period, country, and authors), methodological characteristics (type of intervention, control group and intervention group, type of research, follow-up period and sample), and main results. The data was presented in the form of figures, and descriptive statistics were used, using absolute and relative frequencies.

Thus, the studies were evaluated by two independent evaluators who followed the same search protocol, selecting based on the title, abstract, and full text. If there were any discrepancies, a third reviewer was consulted.

**Figure 1:**
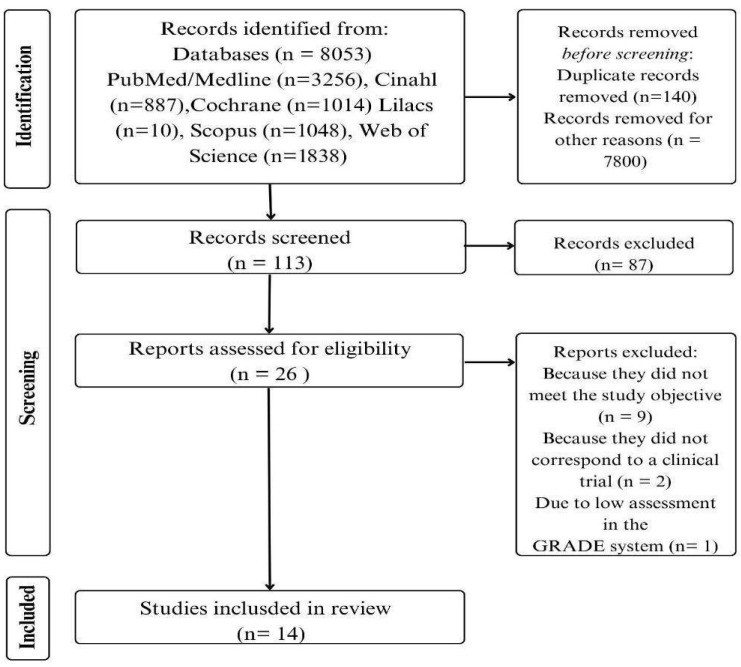
Flowchart of evidence selection. Natal/RN, 2025.

Screening was also carried out by reading the titles and abstracts. The studies were then selected by reading the full text of the articles. The data collected during the search process was stored in a spreadsheet. The eligibility process flowchart is shown in Figure 1.

As for the certainty of the evidence, this was evaluated using the Grading of Recommendations Assessment, Development, and Evaluation (GRADE) system, and the degree of certainty was assessed using Gradepro software https://www.gradepro.org (accessed on 21 June 2021) [14]. An in-depth analysis of the methodological quality of each study was carried out by two independent reviewers. Discrepancies were analyzed by a third reviewer.

**Figure 2:**
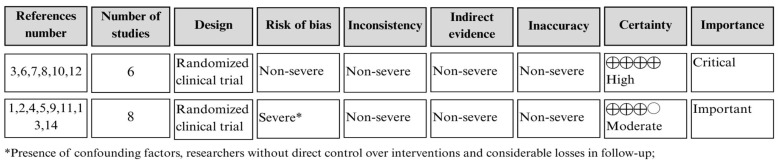
Assessment of the quality of evidence and strength of recommendation of the included studies. Natal/RN, 2025. (reference number in Figure 2: 3,6,7,8,10,12 are the refs. [15,16,17,18,19,20], reference number in Figure 2: 1,2,4,5,9,11,13,14 are the refs. [21,22,23,24,25,26,27,28]).

This tool has been used by systematic reviewers to analyze the quality of evidence. Figure 2 shows the design of the study, its classification in terms of risk of bias, inconsistency, indirect evidence, imprecision, importance, and certainty. The latter classification can be assessed as high (high reliability in estimating the effect), moderate (moderate confidence in estimating the effect), low (limited reliability in estimating the effect), and very low (uncertain reliability in estimating the effect) (XIE GRADE). Studies with low or very low certainty were excluded.

Furthermore, the data on the characterization of the studies is presented in a narrative synthesis shown in Table 1.

## 3. Results

Figure 1 shows the flowchart of the selection process for the studies that make up this systematic review. The final sample included 14 selected articles, all of which were randomized clinical trials.

One study was excluded because it had a low degree of certainty in the GRADE assessment. The study compared the use of two ointments prepared by the pharmacist at the hospital where the study was conducted.

However, women who already had nipple lesions, confirmed by visual examination, were accepted into the control and intervention groups, which could be considered a potential source of confusion in the interpretation of the results, as this was a study focused more on the treatment of nipple-areolar lesions.

In addition, the women were only monitored in part at the hospital after discharge, and they were only given instructions to continue the same practices at home. Thus, the researchers had no direct control over the control group’s non-use of the ointment. The quality assessment of the included studies using GRADE is shown in Figure 2.

Of the final sample, two articles were in the same journal, the *International Breastfeeding Journal*, while the rest were in different journals. Regarding the databases in which the studies were found, 30.7% were in Cochrane, 23.1% were in Scopus and Cinahl, and finally, 7.7% of the articles were identified in the Web of Science, Pubmed/Medline, and Lilacs databases.

Regarding the country of origin, the studies were produced in Turkey (30.7%), Italy, Iran, Brazil (15.4% in each country), Cuba, India, and Australia (7.7% in each country).

With regard to the year of publication, 10% of the articles were published in the last 3 years, 60% in the last 10 years, and 30% before that. Still on the subject of publications, 100% of the studies were published in English.

Prevention strategies that have shown positive results include the use of educational practices, with simulations and demonstrations of breastfeeding techniques in person with a qualified professional (a senior midwife, for example), and also through educational videos. The use of peppermint (in aqueous solution or gel), extra virgin olive oil, honey, guaiazulene ointment, and caseous vernix also emerged with good results. Table 1 details the interventions and results of each study.

**Figure 3:**
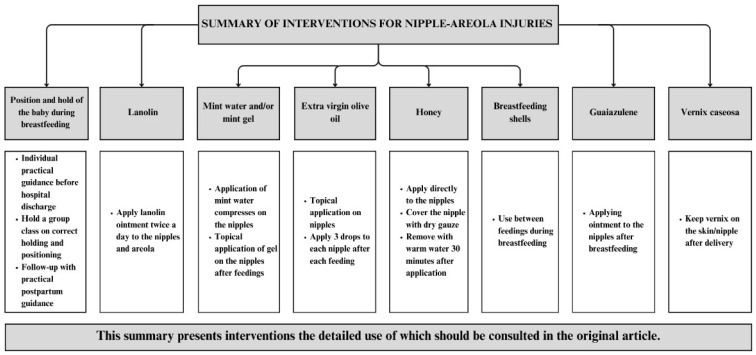
Main interventions on nipple-areolar lesions. Natal/RN, 2025 [15,16,17,18,19,20,21,22,23,24,25,26,27,28].

The main interventions found in relation to the prevention of nipple-areolar lesions that may be helpful during clinical practice are shown in Figure 3.

## 4. Discussion

The main contribution of this systematic review is the grouping of interventions analyzed as effective for the prevention of breastfeeding-related nipple-areolar injuries. In this way, the description of these measures helps clinical practice by providing professionals with quick access to evidence for practice in order to aid decision-making.

Although relevant, topical therapies need to be present alongside educational actions to better help resolve the probable causes of the development of these lesions.

Another important point is the variety of methods for assessing pain, lesions, and the baby’s position during breastfeeding. As for pain, some studies used the Visual Analog Scale, while others used their own questionnaires to measure its intensity.

For nipple-areolar lesions, the Nipple Trauma Index (NTI) was used by one study, while two others assessed them with a ruler and measuring tape: 1–2 mm, mild; 2–9 mm, moderate; >10 mm, severe and/or with a yellowish color in the fissure. Areolar damage was also measured using the same criteria. Many of them did not specify the method of analysis.

Information related to position and latch-on in some contexts was measured using the LATCH scale (latch-on, audible swallow, type of nipple, comfort, and help), which corresponds to latch-on, audible swallow, type of nipple, comfort, and help. Other studies relied on visual analysis of this data by qualified health professionals and the researchers themselves. This information was used to provide guidance on the correct breastfeeding technique.

Correct guidance on positioning and latch-on are shown to prevent nipple-areolar lesions [11]. One study [29] highlights the importance of guidance on breastfeeding techniques, emphasizing the importance of this during prenatal and puerperal care in order to achieve significant results in reducing this damage. In this scenario, the professional nurse plays an important role in prophylaxis and in managing the difficulties encountered by the puerperal woman and the infant [22].

Thus, health education on breastfeeding has been shown to be the most cited approach, with a moderate level of certainty, especially when it is based on guidance, training, and answering questions, and is shown to be effective in reducing the appearance of breast cracks [15,21,22].

Furthermore, the considerable increase in the integration of technological resources in society reinforces the importance of developing innovative means of promoting health education in order to make this process more attractive and dynamic [30]. In this way, the study cited in this paper [23] used video as a visual tool to teach the correct way to breastfeed and prevent complications such as breast fissures, showing positive data in relation to this prevention.

In addition, one of the ways of carrying out health education is counseling and/or training the patient. Among the studies mentioned [21], an education protocol was developed which was applied in separate groups and involved common care, guidance through leaflets, and direct training with demonstration of the appropriate technique. Among these, the option which resulted in the most positive data on the prevention of nipple lesions was training with demonstration. This study presents a moderate level of certainty.

The length of the teaching sessions has also been shown to be relevant in this prevention. One study [22], with a moderate level of certainty, added an additional hour of teaching to the standard education offered by the hospital and found a reduction in nipple trauma, while another study [15], with a high level of certainty, added 30 min to the unit’s routine education and found no statistical difference.

There is also a practice that should be applied from the moment the pregnancy is discovered and after the birth, and that is support. In order to provide quality health education, it is necessary to maintain a support network that includes the patient, the professional, the family, and the partner or friends, as this allows women to feel more secure in the care they receive and to practice the guidelines during their daily lives [15].

As for the care guidelines that should be provided by professionals, they have a direct influence on the success of breastfeeding, with nurses playing an important role in this process [31]. However, another study reinforces the importance of adequate training for these professionals, which is influenced by the context of the practice, respecting the need for changes in the work process and the balance between evidence-based practice and social relevance [32].

This once again confirms the importance of professionals seeking and carrying out constant updating and training during their work. This search for complementary training can and should be encouraged by educational institutions but also by health institutions through the creation of actions, programs, and projects that, in the future, will generate positive results for the healthcare environment, in which professionals can always be looking to develop better care alternatives for the population in general [33].

Considering natural intervention products, those containing peppermint stand out in the literature [16,17,18]. Peppermint belongs to the Mentha genus within the Lamiaceae family and is widely used in the production of infusions, gels, topical formulations, flavorings, and essential oils. Its anti-inflammatory, antioxidant, and analgesic properties have been attributed mainly to menthol, which exerts a local cooling effect and has been associated with pain relief and skin comfort in various clinical contexts [34].

In the context of breastfeeding, peppermint-based preparations have been used primarily for topical application to relieve nipple pain and prevent nipple-areolar lesions. Studies have reported favorable outcomes among women who applied absorbent cotton soaked in peppermint water to the breasts, although these investigations lacked a control group or direct comparison with breast milk application [16,17]. The available studies involving Mentha piperita demonstrated a high level of certainty regarding their reported outcomes; however, methodological limitations should be acknowledged when interpreting these findings.

Furthermore, to date, none of the studies included in this review reported an association between topical peppermint use and reduced milk production. Nevertheless, given the limited number of studies and the absence of outcomes specifically measuring lactation volume, further research is warranted to better explore this relationship. Consequently, the topical use of peppermint for nipple care should be approached with careful clinical judgment and caution, particularly in the absence of robust evidence addressing its effects on milk supply.

In Brazil, the Ministry of Health recommends the application of expressed breast milk to the nipple-areolar complex after each feeding and/or after bathing as a preventive measure against nipple lesions [35]. Breast milk represents a cost-free and readily accessible preventive strategy for breastfeeding women. Although one comparative study suggested lower effectiveness compared to peppermint-based interventions, its established safety profile and accessibility support its continued recommendation within breastfeeding care.

Lanolin is a substance extracted from sheep’s wool and used as a base for the development of cosmetics and pharmacological products.

A study carried out showed that its use is a widespread practice within health services and among professionals who work with breastfeeding, as it has positive repercussions on the care of the lesion from pregnancy to after childbirth. However, although there is proof of its effectiveness in treating nipple-areolar lesions, no evidence was found to support its use for prevention, as shown in the results of this article, with a moderate level of certainty [24,36].

As for guaiazulene (a derivative of plants such as Matricaria chamomilla L., Callis intratropica blue, among others), which is natural from azulene and fat-soluble, it has great anti-inflammatory, anti-infectious, antioxidant, and antifungal properties and is widely used in cosmetics and health care [37]. According to a published study, the compound is also effective in preventing nipple cracks, as highlighted after measuring the effect of guaiazulene ointment and the use of breast milk as an intervention in nipple-areolar lesions in breastfeeding women [27].

When it comes to products based on natural compounds, it is possible to see positive results with extra virgin olive oil, with more than a 97% absence of lesions in the women who used it, in a study with a high degree of certainty. This product has properties that aid healing and prevent inflammation, without showing any adverse effects in the studies [20,25].

Another alternative involves applying honey to the areola. Research involving the application of this substance [19] shows a high level of certainty and describes that honey helps prevent breastfeeding injuries. In this sense, the mechanism of action of honey and its therapeutic actions derive from its antioxidant, antimicrobial, and anti-inflammatory properties. In addition, this product has the ability to stimulate the immune system, cell proliferation, and autolytic debridement, so it can significantly help regenerate skin lesions [38].

However, although some studies included in this review report the use of honey as a topical intervention for nipple-areolar lesions, this practice requires careful consideration. According to the American Academy of Pediatrics, honey intake is contraindicated for infants under 12 months of age due to the risk of infant botulism. Although the application described in the included studies refers to topical use on the mother’s nipple, rather than direct oral consumption by the infant, there is still a potential risk of indirect ingestion during breastfeeding [39].

Therefore, the use of honey on the nipples cannot be universally recommended and should be approached with caution, strict hygiene measures, and professional guidance. Thus, it is noted that future studies should prioritize safer alternatives with well-established safety profiles for both mother and baby.

In relation to vernix caseosa, which is a whitish, oily substance that covers the skin of the newborn at birth, one study showed that it was effective in preventing nipple lesions and relieving pain when compared to breast milk, although care and proper preparation are needed when collecting and storing vernix for use after childbirth [28].

Regarding the evaluation of the use of breastfeeding shells, which are plastic or silicone devices that cover the areola and nipple inside the bra, creating a space for ventilation and avoiding contact with the fabric of clothing, they do not prevent nipple-areolar lesions or pain, as highlighted in the study [26]. Correcting the baby’s grip and positioning is a more satisfactory intervention.

Finally, this research is a means of updating health professionals on how to prevent nipple-areolar lesions in order to help reduce pain and inflammation. Most of the selected studies relate effects from some substance and instructions to the mother, showing that breastfeeding and correct latch-on are essential for preventing injuries and promoting comprehensive care.

One of the limitations found during the search was that most of the studies focused on the treatment of nipple-areolar lesions. In other words, research into prevention is less frequent and needs to be encouraged. In addition, there are few more recent studies on the subject. Most of them cover a period of more than five years. Future research could benefit from expanding search sources to include different databases and journals, thereby covering a more complete spectrum of the available literature.

It is also suggested that other comparative clinical trials be conducted on the use of honey, olive oil, and peppermint, as well as testing other educational strategies using educational technologies, such as realistic simulations, educational podcasts, and teaching using virtual reality, among others.

Although there are some studies addressing this topic, this study adds value in several key aspects, such as its strictly preventive scope and standardized primary outcome (incidence of nipple-areolar lesion), avoiding the heterogeneity of reviews that combined prevention, treatment, and nonspecific pain, updating the body of evidence, incorporating studies published after the time cuts of previous reviews, and a more informative analytical synthesis, with subgroups for potential effect modifiers.

Therefore, our study stands out by synthesizing exclusively the evidence on effective interventions to prevent nipple-areolar lesions, applying methodological rigor (PRISMA, registration in PROSPERO, quality assessment with GRADE) and incorporating recent publications not included in previous reviews. Thus, it contributes by offering clear support for clinical practice and the formulation of healthcare strategies, filling a gap that still exists in the literature.

## 5. Conclusions

It was found that effective prevention interventions for nipple-areolar lesions related to breastfeeding are mainly based on health education and the use of accessible natural products, such as formulations based on peppermint, honey, extra virgin olive oil, and breast milk.

Among these strategies, educational interventions focused on health promotion were described in the vast majority of results because, even if another therapy is chosen, it should be presented in conjunction with guidance and training, teaching the correct breastfeeding positions and the correct latch when sucking breast milk, situations that help prevent nipple-areolar lesions.

The evidence indicates that preventive interventions were applied under specific conditions, considering factors such as formulation, frequency of use, and the postpartum period. Therefore, the decision to use these interventions should take all these points into account (product presentation, frequency of use, postpartum period, among others).

Thus, we reiterate that the absence of standardized protocols reinforces the need for more high-quality research to establish clear, evidence-based guidelines focused exclusively on primary prevention for the use of the interventions found.

Therefore, the importance of these interventions lies in their low cost, feasibility, and potential for home implementation, factors that will influence public health. When supported by adequate professional guidance and contextualized to the needs of lactating women, these strategies can reduce pain, prevent the appearance of nipple-areola lesions, and contribute to the continuation of breastfeeding.

Consequently, strengthening primary prevention interventions represents a relevant and accessible approach to reduce early weaning and promote maternal and child health.

## Figures and Tables

**Table 1:** Characterization of the selected articles. Natal/RN, 2025.

Intervention Type	Control Group	Intervention Group	Follow-Up	Results
Breastfeeding training techniques [21]	Thirty women received routine care; the mothers received breastfeeding services from the neonatal nurse who works at the clinic.	Sixty women received two interventions. Group 1: Thirty women underwent training based on demonstration with a breast model made of fabric, showing the anatomical features of the breast, demonstration of how milk accumulates, how to correct the inverted nipple and the area to be grasped by the baby during sucking, and a specially sewn puppet and a model doll to demonstrate the correct positioning of the baby for breastfeeding. Group 2: Thirty women received training booklets containing information on breastfeeding positions, the importance and benefits of breastfeeding, breast anatomy, breast milk formation, breastfeeding techniques, prevention of breast problems, and breast care using photos. The topics were demonstrated using illustrations and messages encouraging breastfeeding.	14 and 28 days.	The rate of breast lesions was measured by the post-discharge follow-up form, applied by the researcher under the supervision of a health professional. At 14 days, it was 63.3% in group 3, 56.7% in group 2, and 20% in group 1 (*p* = 0.001). At 28 days, the fissure rate was 30% in group 3 and less than 10% in the other two (*p* < 0.005).
Health education on correct position and grip [22]	Thirty-five primiparous women received standard education from the hospital.	Thirty-five primiparous women received standard education offered by the hospital + an additional one-hour teaching session with a senior consultant lactation midwife on correct latch-on, using a doll simulation.	24 h, 4 days, and 6 weeks.	GI women had significantly less pain (*p* < 0.001), according to the visual analog pain scale, and less nipple trauma (*p* < 0.001), according to the Nipple Trauma Index.
30 min counseling session on breastfeeding techniques [15]	One hundred and thirty-seven women received routine guidance on breastfeeding technique.	Seventy-four mothers received routine guidance + reinforcement of breastfeeding technique guidance with demonstration with photos, doll, and breast model.	48 h or 72 h, 7 and 30 days.	There were no statistical differences (*p* > 0.05) between the groups with regard to the frequency of nipple inflammation (cracks, blisters, spots, and/or ecchymoses seen with the naked eye on physical examination), engorgement (excessively full, tender, hardened breasts), and mastitis (fever, malaise, tenderness, reddened breast and antibiotics prescribed), at any assessment point during follow-up.
Video about breastfeeding [23]	Ninety-eight women received the usual care protocol + a recommendation to watch the “Breast is best” video. They were taught to breastfeed sitting upright and to place their babies correctly on the breast following the WHO course.	Ninety women were told to watch the video “Biological nurturing: laid-back breastfeeding for mothers”. They were taught to breastfeed in a relaxed position, with as much contact as possible between the baby’s body and the mother’s chest and abdomen.	At discharge from the maternity ward, on the 7th, 30th, and 120th day after delivery.	At discharge from the maternity ward and on the 7th day after delivery, the IG significantly reduced general breast problems, cracks and inflammation in the nipples. There was no significant difference between the groups in the incidence of mastitis and engorgement. On the 30th day there were no statistical differences between the groups. On the 120th day there was a significant difference in the risk of general breast problems. No other differences were found. The results of the study at 7, 30, and 120 days were collected by telephone, and the participants were asked if there had been any lesions.
Lanolin ointment and health education [24]	Thirty-three women received standard information on breastfeeding.	Thirty-three women received standard GC care + anhydrous lanolin ointment (10 g) for use on the nipple and areola twice a day for six weeks in the prenatal period and postpartum.	8 days.	There were no significant differences between the groups for the prevention of nipple lesions identified on physical examination (*p* = 0.21) and for the prevention of the presence (*p* = 0.61) and intensity of pain (*p* = 0.27) in the nipples, as assessed by the numerical pain scale.
Peppermint [16]	Forty subjects did not use any strategy to prevent lesions.	Forty subjects applied absorbent cotton soaked in peppermint water, obtained from the oil diluted to 0.1%, to the nipple and areola for 20 min twice a day + drying by exposure to air for five minutes.	1, 3 and 5 days.	The mean pain score (scale from 0 to 3) decreased by 0.57 in GI and 0.02 in CG from day 1 to day 5 (*p* < 0.001). There was a mean reduction in the trauma score (scale from 0 to 3) of 0.27 in the IG and 0.02 in the CG (*p* = 0.001) on the 5th day. There was also a reduction in intra-group pain, inflammation, and trauma after 5 days (*p* < 0.005).
Peppermint water [17]	Ninety women applied breast milk to their nipples and areolas after feeding.	Ninety women applied absorbent cotton soaked in peppermint water to the nipples and areolas after feeds, with the recommendation to wash the nipples before the next feed.	4, 8 and 14 days.	The progress of the cases was monitored by telephone interviews with a trained midwife on days 4, 8, and 14 postpartum. In case of nipple or areola cracking and pain, clinical examinations were carried out on the breast, and a measurement scale was applied by a researcher. Women in GI were less likely to have nipple and areola cracks (9%) compared to women who used breast milk (27%; *p* < 0.01).
Peppermint gel [18]	Group 2: Seventy-two mothers received a placebo gel (preparation without peppermint gel) and instructions to wash the area before the next feed.	Group 1: Seventy-two mothers received purified lanolin applied to the nipples and areola after breastfeeding and were instructed to wash the area before the next feed. Group 3: Seventy-two mothers were given peppermint gel and told to wash the area before the next feed.	7, 14 days and 6 weeks.	Overall, nipple cracks were lower in mothers who received peppermint gel than in those who received lanolin ointment or placebo at the end of the study (*p* = 0.01). At 7 days (*p* = 0.144) of follow-up, the results were not significant.
Extra Virgin Olive Oil [25]	One hundred and fifty mothers used drops of breast milk on their nipples after each feed.	One hundred and fifty mothers applied a drop of extra virgin olive oil to the nipple after each feed.	7, 14 and 60 days.	The results were 95% at 7 days, 98% at 14 days, and 99% at 60 days for the presentation of apparent cracks in the nipple (alteration in the integrity of the tissue, by visible macroscopic inspection). The occurrence of nipple cracks was significantly lower in GI (2.7%) than in CG (44.0%) at the end of the study (60 days).
Honey [19]	Twenty women received training by a nurse + breastfeeding guidance with a leaflet on the benefits of breast milk, breastfeeding positions, causes of cracks, and how to avoid them.	Twenty women applied 1 teaspoon of honey three times a day for 30 min for 7 days after breastfeeding + received breastfeeding guidance with an information leaflet.	6 h, 3, 5, and 7 days.	At the end of the 7 days, it was noted that of the mothers in the experimental group, 30.4% had nipple cracks and 76.5% did not. of the mothers in the control group, 69.6% had nipple cracks and 23.5% did not. The difference between the two groups was statistically significant (*p* < 0.01).
Breastfeeding shell [26]	Thirty-three mothers received health education on breastfeeding and clinical demonstration with anatomical breast model, neonatal mannequin, and information leaflet.	Twenty-nine mothers received health education with clinical demonstration, like the control group + use of breastfeeding shells.	7 and 14 days	There was no difference between nipple injury (50.0%) and nipple pain (67.7%) between the groups (*p* = 1). At the third meeting, both groups had favorable parameters for breastfeeding. Only the condition of the breasts was unfavorable (69.4%).
Olive Oil and Breast Milk [20]	Forty women received standardized training on breastfeeding and were instructed to clean the nipple and areola with a piece of wet cotton before each session + after breastfeeding, letting the nipples dry in the open air. No intervention was carried out on or around the nipples of the mothers in this group.	Group 1: Standardized training on breastfeeding + clean the nipple and areola with a piece of wet cotton before each breastfeeding session + after breastfeeding, let the nipples air dry. During the breastfeeding session + after breastfeeding, place three to four drops of breast milk on both nipples. Group 2: Standardized training on breastfeeding + wipe the nipple and areola with a piece of wet cotton before each breastfeeding session. Dry the nipple with a clean towel after breastfeeding and place three to four drops of breast milk on both nipples. Dry the nipple with a clean towel after breastfeeding and apply 3–4 drops of extra virgin olive oil (0.8% acidity) three times a day.	3, 7, 14 days	The number of mothers with nipple pain on the 14th day was significantly lower in the breast milk and olive oil groups (20% and 22.5%, respectively) when compared to the control group (72.5%) (*p* = 0.001). With regard to nipple trauma, on the 7th and 14th days the damage was greater in mothers in the control group (*p* = 0.001) compared to the intervention groups. In addition, it was determined that the cracks that developed on the nipples and areolas of the mothers in the olive oil and breast milk groups were mainly mild, while the mothers in the control group suffered from more severe cracks.
Guaiazulene and breast milk [27]	Group 2: Seventy-seven women applied breast milk after lactation to the surface of the nipple and left to dry for one or two minutes.	Group 1: Seventy-six women applied 0.05% guaiazulene ointment after each breastfeeding period at least four times a day.	15, 30 days	During the study period, the overall incidence of nipple cracks was 31.4%, with 18.4% and 44.2% in the groups treated with guaiazulene and breast milk, respectively (*p* and breast milk, respectively (*p* = 0.001). The magnitude of nipple pain was significantly lower in the guaiazulene group on day 15 (*p* < 0.05) and day 30 (*p* < 0.05) compared to the breast milk group.
Vernix caseosa and breast milk [28]	Thirty-two primiparous women applied breast milk to the nipple and received training on breastfeeding and nipple care.	Thirty-two primiparous women used caseous vernix collected from their own babies after giving birth, as well as receiving training on breastfeeding and nipple care.	Up to 7 days after birth.	On the first day postpartum, the incidence of nipple pain, rashes, and abnormal appearance was similar in both groups (*p* = 0.132, *p* = 0.516 and *p* = 0.132, respectively), and none of the mothers had cracked nipples. However, mothers in the caseous vernix group, on the seventh day after delivery, had significantly less pain (*p* = 0.042), a significant reduction in rashes (*p* = 0.048), significantly greater satisfaction (*p* = 0.023), and no nipple cracks.

## Data Availability

The data that support the findings of this study are available from the corresponding author upon reasonable request.

## Supplementary Materials

The following supporting information can be downloaded at: https://www.mdpi.com/article/10.3390/ijerph23020189/s1, PRISMA checklist.
